# Viability of zooplankton resting eggs in rice field sediment after pesticide applications

**DOI:** 10.3897/BDJ.11.e106418

**Published:** 2023-08-14

**Authors:** Nattaporn Plangklang, Sujeephon Athibai

**Affiliations:** 1 Department of Biology and Applied Taxonomic Research Center, Faculty of Science, Khon Kaen University, Khon Kaen, Thailand Department of Biology and Applied Taxonomic Research Center, Faculty of Science, Khon Kaen University Khon Kaen Thailand

**Keywords:** biodiversity, resting egg, temporary habitat, glyphosate

## Abstract

Many herbicide products are commonly used in agricultural areas to prevent and eliminate weeds. Contamination from these toxicants in water might affect aquatic organisms not only in the active stage, but also in the diapause stage. To test the effect of herbicide on the resting eggs of zooplankton, we prepared two rice fields: one field without the application of pesticides (RF−NPA) and one with the application of pesticides (RF−PA) in a sampling year. We conducted a hatching experiment for 30 days. Twenty–four taxa of zooplankton were found. Sixteen species of these were rotifers, seven species were cladocerans and one taxon was an unidentified nauplius copepod. The species richness of zooplankton between RF–NPA (17 taxa) and RF–PA (16 taxa) was close, but species compositions between RF–NPA and RF–PA were different, indicated by the similarity index of 0.545. Lecanidae was the most diverse family of rotifers in both rice fields with nine species, while Chydoridae was the most diverse family of cladocerans (four species). The total abundance of zooplankton of RF−NPA was higher than RF−PA with 1,897 and 1,286 individuals, respectively. The Shannon–Wiener diversity index (H´) and Pielou’s evenness (*J*) in RF−NPA were higher than in RF−PA. The high species richness of zooplankton in both rice fields occurred on days 18 to 30. On the other hand, the highest abundance was recorded on day 18 for RF−NPA and on day 24 for RF−PA. The non-metric multidimensional scaling (NMDS) demonstrated significant differences in zooplankton community composition between RF–NPA and RF–PA (*p* < 0.05; ANOSIM test). According to the diversity indices, the RF–NPA has more diversity than the RF–PA, which might be a result of herbicide application in the sampling year. This study suggests that the toxicity of glyphosate should be a concern in terms of the biodiversity of rice field ecosystems.

## Introduction

In Southeast Asian countries such as Thailand, a large number of pesticides (over 198,000 tonnes) have been used in rice cultivation (Praneetvatakul et al. 2013). Herbicides (68%) made up the most significant proportion of imported pesticides in 2019, followed by fungicides (14%) and insecticides (12%) (Laohaudomchok et al. 2021). Glyphosate is the most imported herbicide between 2018–2020 and is widely used in Thai agricultural land (Ministry of Agriculture and Cooperatives 2021). Long periods of pesticide applications make rice fields and their surrounding environments severely contaminated (Abdullah et al. 1997). Although rice fields are known as a diverse habitat containing rich zooplankton communities (Heckman 1979, Segers and Sanoamuang 2007), this practice probably causes a decrease in biodiversity within rice field ecosystem, such as zooplankton diversity (Relyea 2005, Plangklang and Athibai 2021, Romero et al. 2022).

Zooplankton have the ability to form resting eggs so they can survive under poor environmental conditions (Radzikowski 2013). Resting egg banks have contributed to the maintenance of local biodiversity and the resilience of the biodiversity of ecosystems (Palazzo et al. 2008, Rosa et al. 2020). Resting stage production can be influenced by predation, competition, drought, low oxygen, nutrients and temperature (Brendonck and De Meester 2003). In addition, low levels of harmful chemicals, such as pharmaceuticals and pesticides, can induce resting egg production in many species of zooplankton (Hanazato 2001, Marcial and Hagiwara 2007). Both pharmaceuticals and pesticides may disrupt the hatching process of zooplankton egg banks (Navis et al. 2013). Herbicides, namely glyphosate, can interfere with the hatching success of zooplankton egg banks under laboratory conditions (Gutierrez et al. 2017, Portinho et al. 2018). The commercial grade of glyphosate at 2.0 – 8.0 mg/l influenced the sexual reproduction of rotifer *Brachionuscalyciflorus* by significantly increasing the mictic rate and resting egg production (Xi and Feng 2004).

The effects of pesticides on hatching resting eggs of zooplankton from sediment have been conducted by applying pesticides directly to sediment under laboratory conditions (Gutierrez et al. 2017, Portinho et al. 2018). However, the hatching of zooplankton egg banks from sediment, collected after herbicide application in the field conditions, still needs to be studied. Field experiments involving pesticides or other environmental chemicals provide a scenario where effects may be explored at the levels of population, community and ecosystem (Walker 2006). We hypothesised that herbicide application probably has effects on the viability of resting eggs and the hatching process of zooplankton in rice fields, leading to reduced species diversity. Thus, to evaluate the hypothesis, we experimentally investigated the species composition of zooplankton in sediment collected from two different rice fields. During the sampling year, the first rice field was planned without the use of herbicides (RF–NPA). The second rice field was treated with glyphosate (RF–PA). Both rice fields revealed high similarity of species occurrences, accounting for 78.61% (Plangklang and Athibai 2021). Moreover, to fulfil the data on the hatching ability of each zooplankton species, we examined the differences in species occurrences at five incubation times.

## Data resources

The data underpinning the analysis reported in this paper are deposited at GBIF, the Global Biodiversity Information Facility, https://doi.org/10.15468/w8h9tn.

## Material and methods

### Study areas

**Figure 1.**
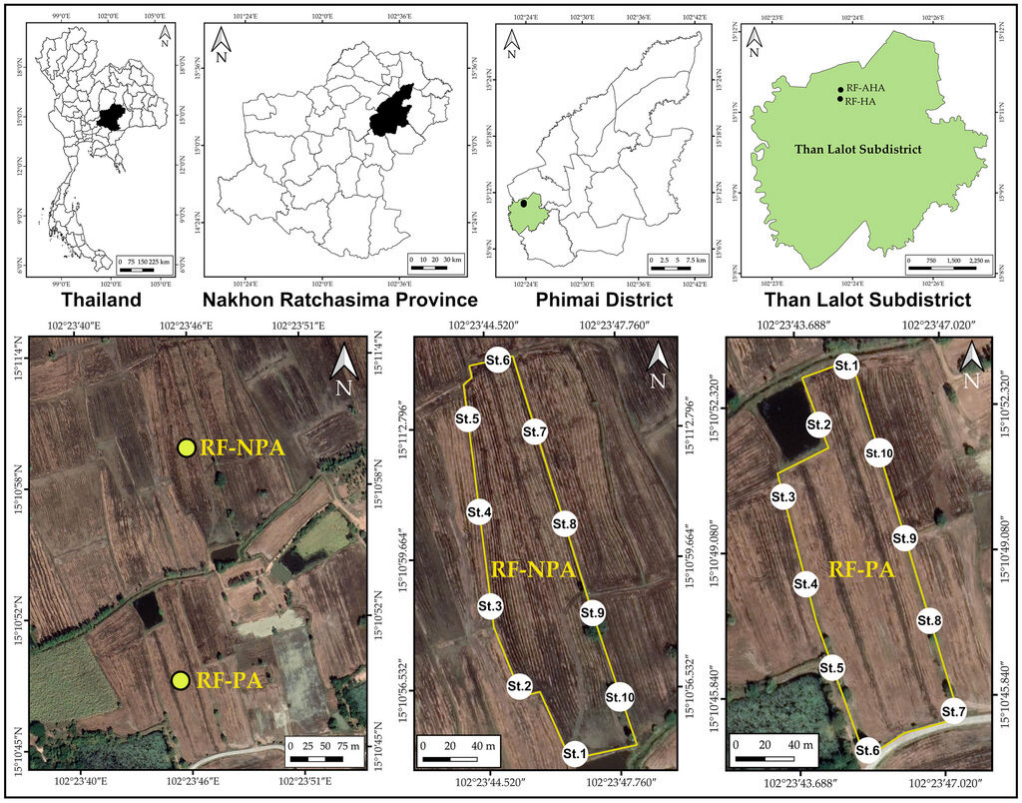
Map of RF–NPA and RF–PA showing ten sampling stations in Than Lalot Subdistrict, Phimai District, Nakhon Ratchasima Province, Thailand. Location maps and sampling stations in satellite view (Google satellite, accessed 30.04.2023), generated from QGIS 3.28.3 (QGIS Geographic Information System. Open Source Geospatial Foundation Project. http://qgis.osgeo.org).

Sediment samples were collected from two rice fields in Ban Non Lukki, Than Lalot Subdistrict, Phimai District, Nakhon Ratchasima Province, north-eastern Thailand (Fig. 1). One was 1.76 ha in size, had not received any pesticides during the year preceding the study (RF−NPA: “Rice Field Non–Pesticide Application”). The second field was 2.08 ha in size and had pesticides applied during the sampling year (RF−PA: “Rice Field–Pesticide Application”). These two rice fields (15°10′55.1″N, 102°23′46.7″E (RF–NPA) and 15°10′45.0″N, 102°23′46.1″E (RF–PA)) are located 146 m above sea level. The distance of RF−NPA is only about 55 m at its closest point to RF−PA. Both RF–NPA and RF–PA were rain-fed rice farming by direct sowing. Rice cultivation occurred only once a year, starting from May to late January and rice was grown approximately 120 days after planting. The rice cultivation process comprised land preparation (May), sowing (June), seedling (late June), fertiliser application (September), pest management (October) and harvesting (late January). Water for both fields came from the same irrigation canal and has been taken from these two fields during rice cultivation. Both rice fields had historical insecticide and herbicide applications (e.g. chlorpyrifos and glyphosate) for at least ten years. In the case of RF−PA, chlorpyrifos (an insecticide, 40% w/v) with 0.48 l/ha of application rate had been used at the seedling stage and glyphosate (an herbicide, 48% w/v) with 1.70 l/ha of application rate was applied at the reproductive stage. Sediments were taken after one week of glyphosate application in the RF–PA.

### Resting egg sampling and hatching procedure

In each of the ten sampling stations, approximately 1 kg of wet sediment was collected from each rice field during the reproductive stage of the rice growing season on 14 October 2018. Then sediment samples were kept in a non-transparent plastic bag at 4°C for one month (Chittapun et al. 2005). After storage, the sediment was dried at room temperature ranging from 28.5 to 30.0°C for one week before the beginning of the experiment. In each of the ten stations per rice field, 90 g of dry sediment was placed in a 1 litre transparent plastic container and 600 ml of distilled water was added. All experimental containers were placed at room temperature (28.65 ± 1.49°C) and 12 hours of daylight exposure in the laboratory. The intensity of light is 1,364.97 ± 458.42 luminosity. Every six days for one month, all supernatant in the container was completely poured out and filtered through a 20 µm mesh size plankton net and 600 ml of fresh distilled water was added into the sediment (Chittapun 2011). Zooplankton samples were preserved by adding 4% formaldehyde immediately. In the case of zooplankton abundance, the number of individuals was probably from both the egg bank and from reproduction due to sampling intervals of six days. Since no food was given to zooplankton during incubation, soil microorganisms may regulate the abundance of zooplankton (Wylie and Currie 1991).

### Zooplankton analysis

All zooplankton samples were identified and counted under an Olympus CH30 compound light microscope. Identification to species level focused on rotifers and cladocerans following the identification keys of Segers (1995), Smirnov (1996), Nogrady and Segers (2002) and research papers (e.g. Sinev (2016)). Photographs of zooplankton were taken under a Nikon Eclipse E200 compound light microscope, equipped with a Nikon Y-TV55 camera.

### Data analysis

The number of species and abundance of each station were normalised using the Shapiro–Wilk test. Owing to a non–normal distribution in the data (*p* < 0.05), the non–parametric statistics were required. The number of taxa and abundance of zooplankton between RF−NPA and RF−PA were compared using the Mann−Whitney U test (df = 9). Pearson’s correlation was used to explore the relationship between the distances amongst sampling points and the similarity of zooplankton species in the rice fields. These three statistics were conducted in IBM SPSS Statistics for Windows (version 28.0; IBM Corp., Armonk, NY, USA).

The Sørensen−Dice similarity index (*Cs*) (Magurran 2004) was applied to express the similarity in species composition of zooplankton between rice fields (RF−NPA and RF−PA) and between sampling stations within the rice field. The Shannon–Wiener diversity index (H´) (Shannon and Weaver 1949) and Pielou’s evenness index (*J*) (Krebs 1999) were used to measure species diversity and species-abundance distributions of zooplankton community in the rice field sediments.

The species accumulation curve (Oksanen et al. 2019) and four non-parametric estimators, including Chao1, Jackknife1, Jackknife2 and Bootstrap (Colwell and Coddington 1994) were used to estimate species richness in each rice field sediment, based on species abundance data. In addition, non-metric multidimensional scaling (NMDS) was employed to cluster zooplankton community in RF–NPA and RF–PA amongst five sampling times, based on Bray-Curtis dissimilarities. Analysis of similarities (ANOSIM) was performed to test differences in zooplankton community composition in RF–NPA and RF–PA (Oksanen et al. 2019). These analyses were performed using the package ‘vegan’ in RStudio (version 3.6.1, RStudio, Boston, MA, USA).

## Results

### Species richness of zooplankton

**Figure 2.**
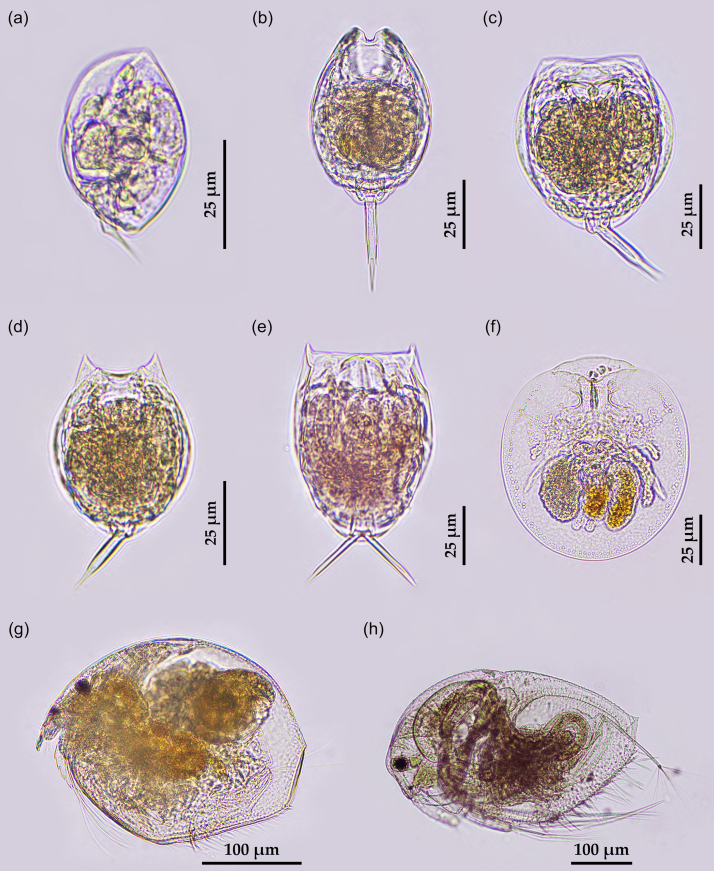
Some rotifers (a–f) and cladocerans (g–h) a: *Colurellaobtusa* (Gosse, 1886); b: *Lecanebulla* (Gosse, 1851); c: *Lecaneclosterocerca* (Schmarda, 1859); d: *Lecanehamata* (Stokes, 1896); e: *Lecanesignifera* (Jennings, 1896); f: *Testudinellapatina* (Hermann, 1783); g: *Ephemeroporusbarroisi* (Richard, 1894) and h: *Macrothrixtriserialis* Brady, 1886.

A total of 24 taxa of zooplankton were found. Sixteen species of these corresponded to rotifers, seven species were cladocerans and one taxon was nauplius of copepods. The species list of zooplankton is shown in Table 1. Some zooplankton are shown in Fig. 2. Although the number of taxa of the zooplankton between RF–NPA and RF–PA was close, accounting for 17 and 16 taxa, respectively, the Sørensen−Dice similarity coefficient is 0.545.

The species richness of rotifers hatched from the RF−NPA sediment (11 species) was lower than the RF−PA (13 species). Lecanidae with nine species accounting for 56.25% of the total richness of rotifers, was the most diverse family in both rice fields, followed by Lepadellidae and Notommatidae, each with two species. The species richness of cladocerans in the RF−NPA was higher than the RF−PA with five and three species, respectively. The most diverse family was Chydoridae with four species (57.14% of total richness of cladocerans), followed by Ilyocryptidae, Macrothricidae and Moinidae (one species in each family) (Table 1). However, the result of the Mann–Whitney U test demonstrated there was no significant difference in species richness of zooplankton between RF−NPA and RF−PA (Z = –1.268, *p* = 0.205).

The species richness of zooplankton in both rice fields tended to increase over incubation periods (Table 1). In RF−NPA, zooplankton richness began increasing at day 12 with eight species. The maximum number of species was reached on day 24 (14 species). After day 24, zooplankton richness showed a small decrease. On the other hand, the number of species of zooplankton in RF−PA increased at day 12 with seven species and maximum richness was reached at day 30 (10 species). The number of zooplankton taxa in RF–NPA and RF–PA comprised seven and six taxa, respectively, at the first incubation time (day 6). A total of 14 of 17 taxa for RF–NPA and 13 of 16 species for RF–PA were found within 18 days of the incubation period. In addition, 16 zooplankton species in RF–PA completely hatched within 24 days, while the occurrences of zooplankton in RF–NPA were found up to day 30.

The highest species richness of rotifers in RF−NPA was reported on day 24 with 10 species, whereas the maximum number of species for RF−PA was noted on days 18 and 30 (eight species, each one) (Table 1). Lecanidae was the most diverse family amongst the five incubation periods. Only *Lecanehamata* was found in every incubation period of both rice fields (Table 1).

**Figure 3.**
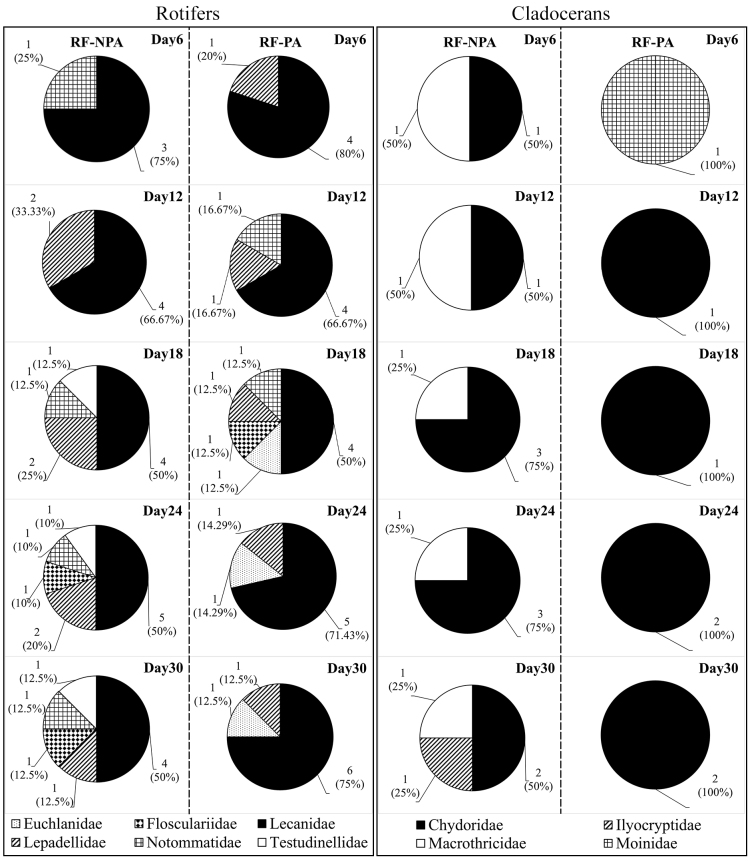
The proportion of the families of rotifers and cladocerans hatching from RF−NPA and RF−PA sediments at different incubation times.

The highest species richness of cladocerans for RF−NPA was recorded on days 18, 24 and 30, with four species each, while the greatest number of species for RF−PA was found on days 24 and 30, with two species each (Fig. 3). On day 6, there were differences in species occurrences of cladocerans between RF−NPA and RF−PA. *Leberisdiaphanus* and *Macrothrixtriserialis* were found in RF−NPA sediments; by contrast, *Moinamicrura* was recorded only from the latter rice field (Table 1). In addition to copepods, one unidentified nauplius was found only in RF–NPA on day 6.

**Figure 4.**
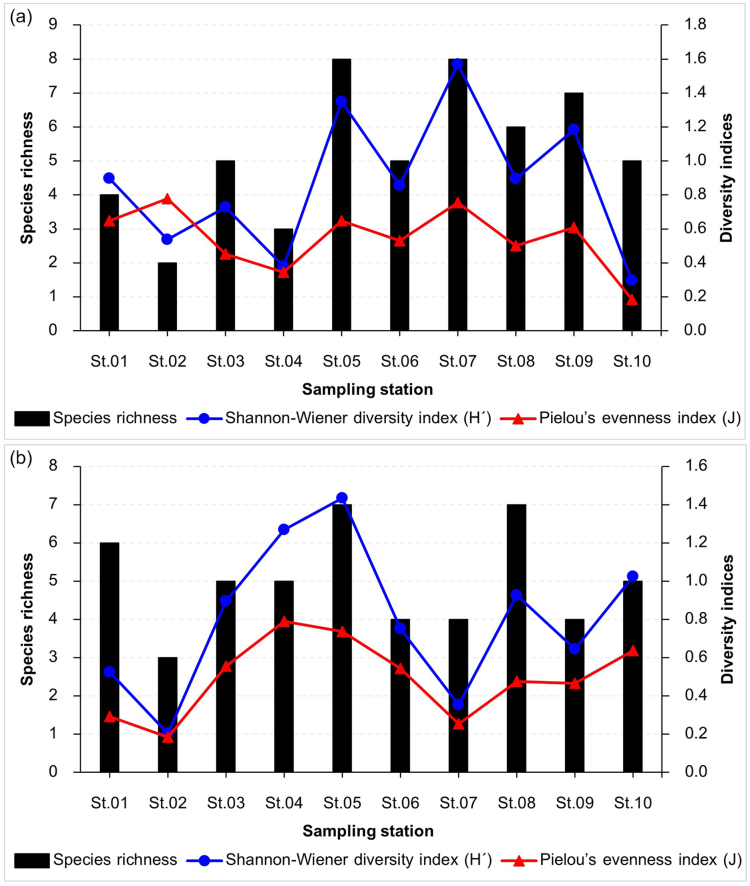
Species richness of hatched zooplankton, Shannon–Wiener diversity index (H´) and Pielou’s evenness index (*J*) amongst ten sampling stations of RF–NPA (a) and RF–PA (b).

The total number of species per sampling station in RF–NPA ranged from 2–8 species. Eight species have been identified at stations 5 and 7, for the greatest species total (Fig. 4a). Station 5 had the greatest average species richness, with 4.00 ± 2.00 species (Fig. 7a). On the other hand, the total species richness in RF–PA ranged between three and seven species. Stations 5 and 8 had the maximum species richness, with seven species each (Fig. 4b). The highest mean value of the number of species was 3.75 ± 0.96 species which was noted in station 4 (Fig. 7c).

The greatest Shannon–Wiener diversity index (H´ = 1.521) and Pielou’s evenness index (*J* = 0.536) were recorded in RF–NPA (Table 2). The highest diversity index (1.570) and evenness index (0.777) for RF–NPA were recorded from stations 7 and 2, respectively (Fig. 4a). On the other hand, the maximum values of diversity index (1.433) and evenness index (0.789) for RF–PA were found at stations 5 and 4, respectively (Fig. 4b) Suppl. material 1.

**Figure 5.**
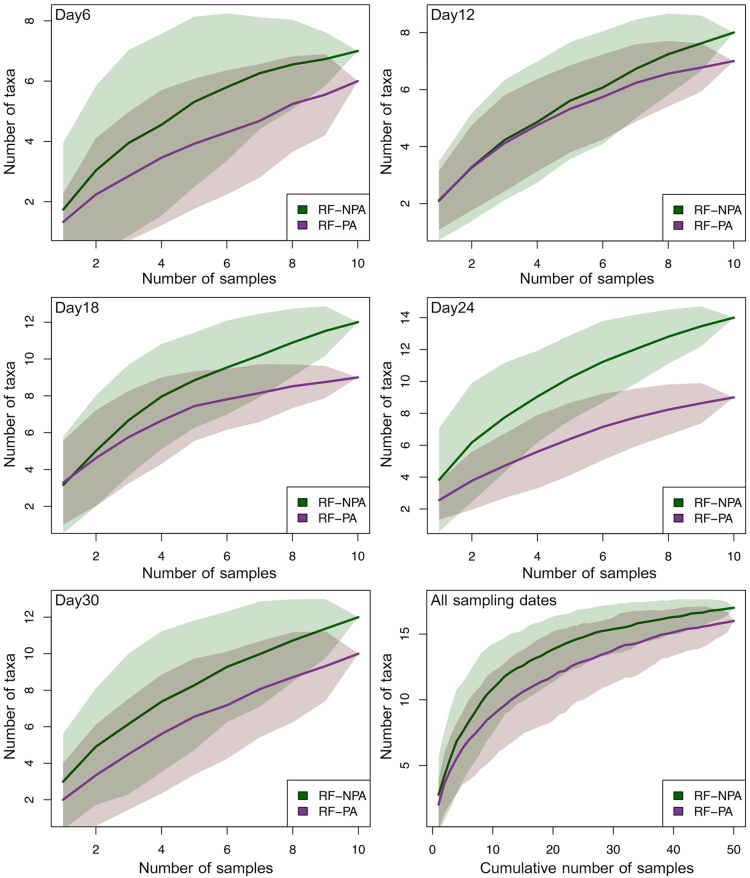
Comparison of species–accumulation curves of zooplankton for RF–NPA and RF–PA sediments amongst five incubation times.

According to the species accumulation curves, both rice fields had a similar pattern with an increase in the number of species of hatched zooplankton. However, the steepness of the accumulation curve was initially greater in RF–NPA than in RF–PA, resulting in a higher number of species in RF–NPA (Fig. 5). Considering the curves in each sampling date, the species richness of emerged zooplankton in RF–NPA was greater than RF–PA throughout five incubation times (Fig. 5).

The estimation of the number of zooplankton species in RF–NPA and RF–PA revealed that three estimators (Chao1, Jackknife1 and Jackknife2) extrapolated the number of zooplankton taxa in RF–PA greater than that in RF–NPA. On the other hand, bootstrap provided the estimated value for RF–NPA being higher than RF–PA. Jackknife2 revealed the lower estimated number of taxa for RF–NPA (14.24 species) compared to the number of observed taxa (17 species) (Table 3).

The highest similarity index for RF–NPA was 0.800 which related to stations 7 and 9, but the lowest value was only 0.154 which corresponded with stations 3 and 5. In RF–PA, the maximum value of the similarity index (0.889) was recorded from stations 4 and 6, while the minimum value (0.182) was from stations 7 and 8 (Suppl. material 2). However, the result of Pearson’s correlation revealed the distance between sampling points did not influence the similarity of zooplankton species in the rice fields (r = –0.067, *p* = 0.792).

### Abundance of zooplankton

**Figure 6.**
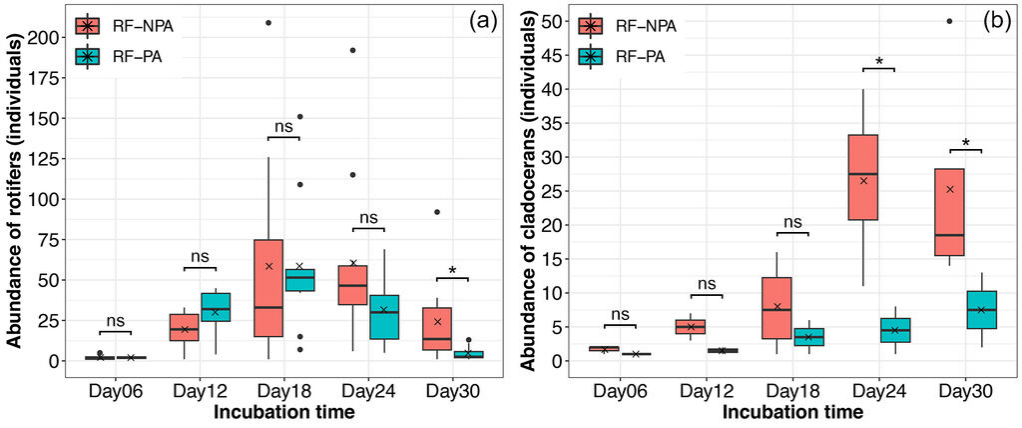
Total abundance of rotifers (a) and cladocerans (b) at different incubation periods. An asterisk (*) indicates statistically significant differences (*p* < 0.05) between RF–NPA and RF–PA. A cross (×) on the boxplot indicates the mean value.

The total number of zooplankton in RF−NPA was higher than in RF−PA with 1,897 and 1,286 individuals, respectively. The total abundance of rotifers in RF–NPA was significantly greater than that in RF–PA (Z = –2.228, *p* = 0.026) only on day 30 with 34.57 ± 17.18 and 5.14 ± 2.81 individuals, respectively (Fig. 6). Rotifers showed a low number of individuals at the first incubation time (day 6); thereafter, the abundance began increasing at day 12 and significantly decreased on day 30. The greatest total number for RF−NPA was noted on day 24 (605 individuals), while the highest abundance for RF−PA was reported on day 18 (586 individuals). *Lecanebulla* (937 individuals) was the most abundant rotifer species in RF−NPA sediment. By contrast, the highest abundance species for RF−PA were *Lecaneclosterocerca* (758 individuals). *L.bulla* (26 individuals) and *L.hamata* (249 individuals) in RF−PA had a lower total number of individuals than these species in RF−NPA, with 937 and 517 individuals, respectively. On the other hand, *Colurellaobtusa* (67 individuals), *L.closterocerca* (758 individuals) and *L.tenuiseta* (113 individuals) in RF–PA showed a higher total abundance than in RF–NPA, which were 16, 85 and 50 individuals, respectively. *Euchlanisincisa* (20 individuals) was found only in RF–PA (Table 1).

For cladocerans, the total number of individuals in RF−NPA on days 24 and 30, with 26.50 ± 12.23 and 25.25 ± 16.76 individuals, respectively, were significantly higher than RF−PA, which had 4.50 ± 4.94 and 6.50 ± 7.78 individuals, respectively (Z = –2.141, *p* = 0.032 for day 24; Z = –2.121, *p* = 0.034 for day 30) (Fig. 6b). In RF−NPA, cladocerans showed a low number of individuals (four individuals) on day 6, reaching their maximum abundance on day 24 (106 individuals) and decreasing slightly on day 30 (101 individuals). In contrast to RF−NPA, RF−PA sediments revealed low abundance of cladocerans throughout five incubation times (day 6 to day 30). At the first incubation period (day 6), the abundance of cladocerans in RF−PA was low, with only one individual and reached the highest abundance during the last incubation time (day 30), with 13 individuals. *Ephemeroporusbarroisi* was the most abundant cladoceran species in RF−NPA (100 individuals) and disappeared in RF−PA; whereas *Karualonakarua* was the most abundant species in RF−PA (28 individuals) and absent in RF−NPA (Table 1). *Le.diaphanus* showed a delay in hatching (on day 24) with lower abundance in RF−PA (only two individuals), compared to the number of individuals in RF−NPA (70 individuals). Moreover, *Ma.triserialis* was absent amongst five incubation times of RF−PA, while this species had a high abundance in RF−NPA with 74 individuals (Table 1). Only one individual nauplius larva of copepods was recorded from RF–NPA on day 6.

**Figure 7.**
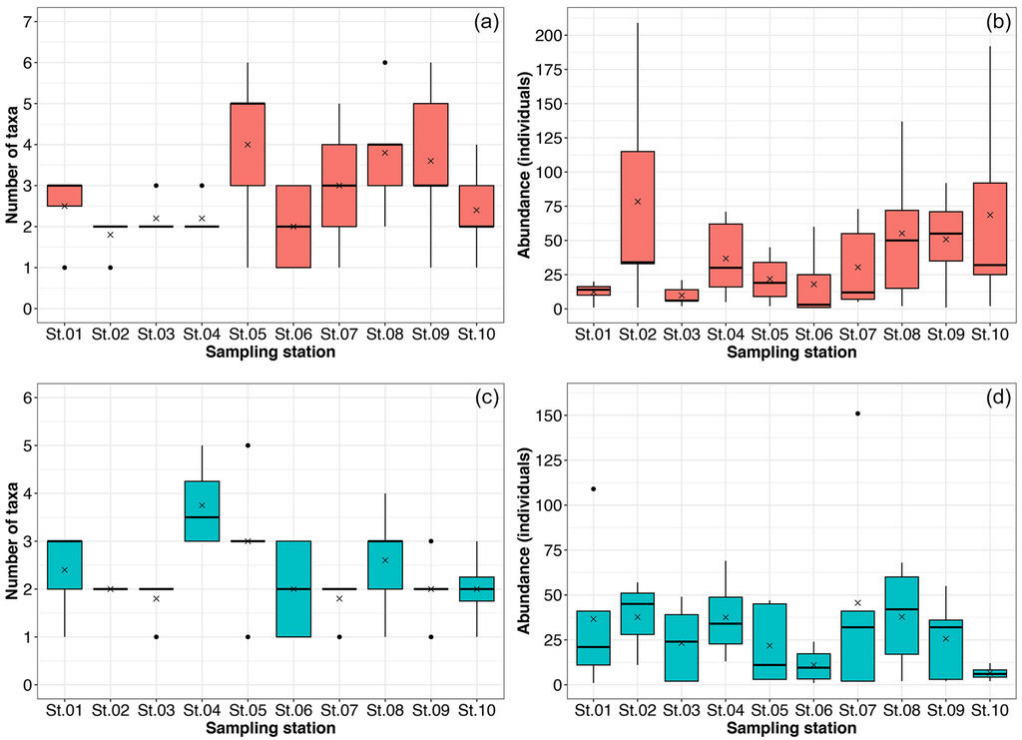
The number of taxa and abundance of zooplankton in RF–NPA (a, b) and RF–PA (c, d) amongst ten sampling stations. A cross (×) on the boxplot indicates the mean value.

Zooplankton abundance in RF–NPA ranged between 1 and 209 individuals. Station 2 revealed the greatest abundance of zooplankton, with 209 individuals (Fig. 7b). By contrast, the total number of zooplankton in RF–PA ranged from 1 to 151 individuals. The highest number of individuals was recorded at station 7, which was 151 individuals (Fig. 7d).

### Community composition of zooplankton

**Figure 8.**
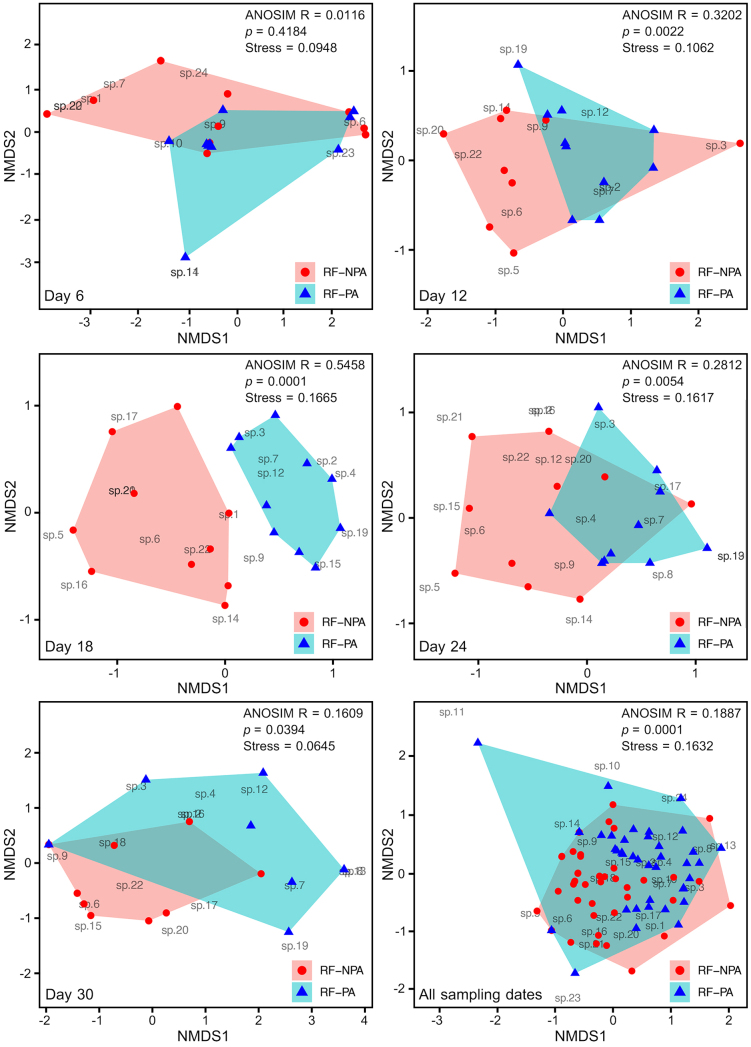
Non-metric multidimensional scaling (NMDS) ordinations representing the similarity of zooplankton community between RF–NPA and RF–PA sediments amongst five sampling times.

The NMDS analysis demonstrated distinct groupings and the dissimilarity between RF–NPA and RF–PA zooplankton communities, indicated by ANOSIM test results with the R statistic value and the corresponding significance level (*p*–value) (Fig. 8). A significant difference in the community structure was examined between RF–NPA and RF–PA in the combination of all sampling dates due to an R statistic value of 0.1887 and a significance level of 0.0001. The separation of communities by the occurrence of seven species, including *Cephalodellaforficula*, *Lecanearcula*, *Testudinellapatina*, *E.barroisi*, *Ilyocryptusspinifer*, *Leydigiaciliata* and *Ma.triserialis* only in RF–NPA and the existence of seven species, namely *E.incisa*, *L.haliclysta*, *L.inopinata*, *L.signifera*, *L.undulata*, *K.karua* and *Moinamicrura* only in RF–PA was marked. Comparing each sampling date, significant differences in the zooplankton community between RF–NPA and RF–PA were observed on days 12 (R statistic value = 0.3202, *p* = 0.0022), 18 (R statistic value = 0.5458, *p* = 0.0001), 24 (R statistic value = 0.2812, *p* = 0.0054) and 30 (R statistic value = 0.1609, *p* = 0.0394) (Fig. 8). On day 12, a significant difference in zooplankton composition was indicated by the appearance of *L.arcula*, *Lepadellapatella*, *Le.diaphanus* and *Ma.triserialis* only in RF–NPA community and occurrences of *C.gibba* and *L.closterocerca* only in the RF–PA community. On day 18, a significant difference in zooplankton community between RF–NPA and RF–PA related to the existence of *C.forficula*, *L.arcula*, *Lep.patella*, *T.patina*, *E.barroisi*, *Le.diaphanus*, *Ley.ciliata* and *Ma.triserialis* only in RF–NPA and the appearance of *C.gibba*, *E.incisa*, *L.closterocerca*, *S.spinosa* and *K.karua* only in RF–PA. On day 24, the zooplankton community structure between RF–NPA and RF–PA was different which corresponded to the occurrence of *C.gibba*, *L.arcula*, *L.bulla*, *Lep.patella*, *S.spinosa*, *T.patina*, *E.barroisi*, *Ley.ciliata* and *Ma.triserialis* only in RF–NPA and the appearance of *E.incisa*, *L.haliclysta*, *L.undulata* and *K.karua* only in RF–PA. On day 30, the occurrence of *C.gibba*, *S.spinosa*, *T.patina*, *E.barroisi*, *I.spinifer* and *Ma.triserialis* only in RF–NPA and the presence of *E.incisa*, *L.haliclysta*, *L.undulata* and *K.karua* only in RF–PA led to a significant difference in zooplankton community between RF–NPA and RF–PA. The similarity index values between RF–NPA and RF–PA for day 12, Day 18, day 24 and day 30 were 0.533, 0.381, 0.434 and 0.545, respectively. However, there was not a significant difference in zooplankton composition between RF–NPA and RF–PA on day 6.

## Discussion

The hatching of zooplankton resting eggs plays an important contribution to zooplankton diversity in rice fields. The existence of zooplankton communities not only comes from irrigation canals, but also from hatching of resting eggs remaining within rice fields (Chittapun 2011). Resting egg banks maintain a high species diversity in communities and sustain the genetic variability of populations (Brendonck and De Meester 2003). Applications of pesticides and other harmful chemicals can influence different pathways of zooplankton; these compounds have an impact on the mortality of embryos in resting eggs, hatching characteristics, growth and reproduction, as well as resting egg production (Navis et al. 2013).

The diversity and abundance of resting egg zooplankton collected from a rice field with pesticide application (RF–PA) in comparison to a non-treated pesticide rice field (RF–NPA) were conducted in the present study, based on incubation sediment for one month. The number of hatched zooplankton taxa in RF–NPA (17 taxa) and RF–PA (16 taxa) was close, whereas species composition between two water bodies is different (54.50% of the similarity). This was also supported by the NMDS ordinations, which revealed the significant differences in zooplankton composition between RF–NPA and RF–PA. The ordinations performed with a stress values range of 0.06 – 0.16, which were in the “good” to “usable picture” criteria range (Dexter et al. 2018). Although RF–NPA and RF–PA are located closely together by their distance (55 m), the resting egg assemblages between two rice fields are distinctive. Our results differed from Ismail and Zaidin (2015), who found that zooplankton composition found in two sampling sites located in the same irrigation canal, has a high value of the similarity coefficient, with more than 0.80. Likewise, two water bodies situated in the same watershed show a high similarity of rotifer occurrences, accounting for 91% of the similarity (Plangklang and Athibai 2019). The extrapolated number of zooplankton taxa in RF–PA was greater than that in RF–NPA based on Chao1, Jackknife1 and Jackknife2. This is because a large number of singletons occurred in RF–NPA, with 44 in comparison to RF–PA which had 31. The species richness estimates are markedly influenced by species with low frequencies of records (Demétrio and Silvestre 2013). Shen et al. (2023) found that, although the Chao1 estimator is applied to reduce bias in estimates of species richness, singleton species have a strong effect on predicted values. The shapes of accumulation curves appeared to differ amongst the five incubation times, which is influenced by the number of species and the proportional number of singletons in the sample (Thompson et al. 2007).

The zooplankton resting egg bank in RF–NPA and RF–PA demonstrates 25.80% and 25.50% similarity to active zooplankton in the water body itself which has been reported by Plangklang and Athibai (2021). This result is in accordance with Araújo et al. (2013), who revealed that a low concordance between active and dormant communities is found in temporary environments. Battauz et al. (2014) have stated that the diversity of hatched zooplankton resting eggs was lower than the active community, probably depending on the number of samples and stimuli specific to the taxon. According to 16 taxa for RF–NPA and 16 taxa for RF–PA identified to species level, one species, namely *Ley.ciliata* and four species, including *E.incisa*, *L.undulata*, *Le.diaphanus* and *M.micrura*, are found only in sediment samples of RF–NPA and RF–PA, respectively, but those are absent in active zooplankton communities. It is noteworthy that the appearance in the resting stage of those species indicates a life strategy to escape from unfavourable conditions (Aránguiz-Acuña and Serra 2016).

Chlorpyrifos and glyphosate have been used in both RF–NPA and RF–PA for at least ten years. The half-lives of chlorpyrifos and glyphosate in fields vary from a few days to several months, the mean half-lives of chlorpyrifos and glyphosate in sediment being 38 and 47 days, respectively (Giesy et al. 2014, Gandhi et al. 2021). However, chlorpyrifos was not detected in water samples of RF–PA because this chemical is used at the seedling stage of rice (90 days before sampling) according to Plangklang and Athibai (2021). Chansuvarn and Chansuvarn (2018) and Romero et al. (2022) reported that the levels of glyphosate in the water and soil of rice fields are relatively low, with 0.90 µg/l and 0.26 µg/kg, respectively. Applications of glyphosate and chlorpyrifos in the past and use of chlorpyrifos in the sampling year seem to have no impact on the species richness and abundance of zooplankton in rice fields due to their short half-lives (38 days for chlorpyrifos and 47 days for glyphosate) (Giesy et al. 2014, Chansuvarn and Chansuvarn 2018, Gandhi et al. 2021, Romero et al. 2022). However, the application of glyphosate in the sampling year probably affected the viability of zooplankton resting eggs in RF–PA. Since glyphosate was applied in RF–PA one week before sampling, this herbicide might remain in the environment based on its half–life (Gandhi et al. 2021).

Although there were no significant differences in numbers of taxa of hatched zooplankton between RF–NPA and RF–PA, differences in the species composition and abundance of hatched zooplankton between both rice fields were found. Portinho et al. (2021) found that the species richness of rotifer in the sediment treated with 0.44 and 0.89 mg a.i./l glyphosate has significantly decreased compared to the incorporated group. Glyphosate can affect the hatching dynamics of zooplankton because this substance has the characteristics to pass through or relate to the membranes covering the resting eggs (Gutierrez et al. 2017). Accordingly, fenoxycarb insecticide can accumulate in resting eggs, affecting the hatching and development of *Daphniamagna* (Navis et al. 2015). This suggests that only one practice was sufficient for causing adverse effects on both active zooplankton communities and resting egg banks in sediment. The toxicity of pesticides on non-target organisms in rice fields contributes to several factors, including chemical properties, rate and frequency of applications, nature of the impact, environmental conditions and methodology (Simpson and Roger 1995).

The abundance of zooplankton between RF−NPA and RF−PA is different. Both rotifers and cladocerans in RF−NPA appeared higher in abundance in comparison to RF−PA, especially the number of individuals of cladocerans from the RF−PA sediment which showed less abundance throughout five incubation times. It seems likely that glyphosate applications might be a causative factor that disrupts resting egg production and the dormancy in resting eggs of some zooplankton in rice fields. This evidence is from the abundance of rotifers and cladocerans in RF–PA, with cladocerans having a lower number of individuals than rotifers. This result indicated that cladocerans were more sensitive to glyphosate than rotifers. Romero et al. (2022) revealed that cladocerans are the most sensitive group amongst zooplankton in rice fields which have agrochemical application (herbicides: bentazone, clomazone, glyphosate; insecticide: imidacloprid; fungicide: tebuconazole). Cladoceran species also appear different in their sensitivity to pesticides (Hanazato and Kasai 1995, Hayasaka et al. 2012).

The number of individuals of *L.bulla* and *L.hamata* in RF−PA were low compared to RF–NPA. It seems that these two rotifers suffer from glyphosate application. Although there was no evidence on the sensitivity of *L.bulla* and *L.hamata* to glyphosate, *L.bulla* and *L.hamata* have been reported as the most sensitive to copper (Cu) and lead (Pb) (Rico-Martínez et al. 2013). By contrast, *C.obtusa*, *L.closterocerca* and *L.tenuiseta* showed a higher abundance in RF–PA than in RF–NPA. In addition, *E.incisa* and *K.karua* had high abundance only in RF–PA. Those zooplankton species can likely tolerate glyphosate. *L.closterocerca* and *E.incisa* have been reported as tolerant taxa to the fungicide carbendazim (Daam et al. 2010). *Alonaguttata* which is in the same family as *K.karua*, is more tolerant to Faena™ (glyphosate formulation) than *Daphniaexilis* (*Lares et al. 2022*). Furthermore, one cladoceran species, *Le.diaphanus*, demonstrated a delay in the hatching in RF−PA. This result is similar to the action of fenoxycarb (insecticide) on the hatching characteristics of *Daphniamagna* dormant eggs (Navis et al. 2013). Brendonck and De Meester (2003) reported that delayed hatching is found in many zooplankton species. This is considered a strategy to survive, maintaining their high genetic and species diversity. Furthermore, the sensitivity of each zooplankton group after being exposed to pesticides is different. Rotifers are more tolerant than cladocerans to many pesticides (Chang et al. 2005). According to Gutierrez et al. (2017), glyphosate could be disrupting the hatching dynamics of zooplankton egg banks, which leads to a significant decrease in species diversity. Similarly, Portinho et al. (2018) found that formulated herbicides containing 2,4−D and glyphosate and their mixture which were applied directly to sediment showed a significant reduction in the total abundance and species richness of rotifers. Glyphosate and the mixture were causes of a decrease in the total abundance and total taxon richness of zooplankton. In contrast, individual herbicides and their mixture did not affect the abundance and species richness of cladocerans and copepods.

Only one nauplius of copepod was found in RF−NPA sediment at the first incubation time (day 6), but it was not present in RF−PA. Although there is no evidence of resting egg production of eight diaptomids and three cyclopoid copepods which are recorded in the report of Plangklang and Athibai (2021), some diaptomid copepods, *Skistodiaptomuspallidus* can produce resting eggs (Dowell 1997). However, Fantón et al. (2020) found that 0.38 mg/l of glyphosate causes an increase in the development times of freshwater copepod nauplii, *Notodiaptomuscarteri*. Exposure to 0.81 mg/l glyphosate also interrupts the copepod development during the larva stage entering the adult stage by inhibiting the growth of the first copepodite stage. That concentration led to an increase in both antioxidant enzymes GST and SOD activity in adult females.

*Lecane* was the most diverse genus of rotifers with nine species, accounting for 56.25% of the total richness of rotifers. The high diversity of *Lecane* confirms that all recorded lecanid species are widely distributed in the tropical region. *L.bulla*, *L.closterocerca*, *L.hamata* and *L.tenuiseta* are cosmopolitan, whereas *L.arcula*, *L.haliclysta*, *L.inopinata*, *L.signifera* and *L.undulata* exhibit as tropicopolitan species (Segers 1996). *L.closterocerca* has also shown the highest abundance in RF−PA sediments. It is noteworthy that some species in the genus *Lecane* have more tolerance ability under adverse conditions than other species. This is similar to the case of the diversity of zooplankton under straw-burning sediment, only three species in the genus *Lecane*, including *L.bulla*, *L.luna* and *L.tenuiseta* occurring in straw burning conditions (Chittapun 2011). In monogonont rotifers, the outer (S1) and inner (S2) membranes as well as the appearance of mucopolysaccharide contribute to protecting the embryo in resting eggs from adverse conditions (Denekamp et al. 2010). The absence of the extra-embryonic space in the resting eggs of *L.bulla* might have contributed to reducing the accumulation of harmful chemicals in the eggs (Guerrero-Jiménez et al. 2020).

*M.micrura* was found only on day 6 of the RF−PA sediment. Our findings agree with the results of Santangelo et al. (2011), who found that the hatching time of *M.micrura* ranges from 5 to 9 days. Furthermore, just one individual of *M.micrura* was found, which may relate to the observation that it seems more sensitive to glyphosate than other cladocerans. The 48-h LC_50_ of *M.micrura*, *Ceriodaphniadubia* and *Daphniamagna* is 3.04, 5.70 and 146 mg/l, respectively (Iwai et al. 2011, Rico-Martínez et al. 2012). Regarding the resistance of resting eggs of *M.macrocopa*, concentrations up to 60–70 g/l of copper, cadmium, zinc and nickel did not affect the viability of resting eggs of this species over a 30-days exposure (Oskina et al. 2019). It seemed that the occurrences of zooplankton probably depend on the viability and resistance of resting eggs to unfavourable conditions, as well as the incubation period (Brendonck and De Meester 2003, Chittapun 2011). Similarly, *Ley.ciliata* was reported as a rare species in the present study by only two individuals emerging from RF−NPA at day 18 and day 24 of the incubation periods. This was confirmed by Iglesias et al. (2016), who stated that Ley.cf.striata is only recorded from hatching sediment and is absent in the active community in waterbodies. Some species, such as *I.spinifer*, seemed to require a longer time to hatch because this species was found only at day 30 of RF−NPA sediment. The hatching time of this species differs from Eskinazi-Sant’Anna and Pace (2018), who reported the presence of *I.spinifer* in the sediment of a temporary lake; Coutos Lake in Brazil from day 10 to day 30 of incubation times and has a high abundance, with more than 100 individuals.

Since zooplankton were sampled every six days, rotifers and cladocerans can reproduce during incubation. Therefore, the abundance was probably from both the egg bank and reproduction due to long sampling intervals over 3 days (Vendramin et al. 2021). However, there was no feeding throughout incubation periods and food deficiency in the hatching environment reduced the reproduction performance of rotifers and cladocerans (Weithoff 2007). It could be that food availability in sediment has contributed to differences in the abundance of zooplankton between RF–NPA and RF–PA. Food availability is one of the most significant factors influencing the population density of zooplankton (Vanni 1987). A significant decrease in the fecundity of rotifers and cladocerans caused by food limitation and starvation has been reported (Duncan 1989, Yoshinaga et al. 2003). Microorganisms in sediment seem to be food sources for zooplankton in this study. Bacteria and cyanobacteria as substantial food sources for zooplankton (Work and Havens 2003) and are major components in microbial communities in rice fields (Song et al. 2005). The application of glyphosate probably affects the composition and abundance of food items. Pérez et al. (2007) reported that glyphosate has a direct effect on microbes by changing their community assemblages. Likewise, glyphosate-based herbicide (Roundup) affects bacterial and archaeal communities in soil, with decreases in diversity and changes in community structure (Mesquita et al. 2023). Therefore, the application of glyphosate in rice fields might have an indirect effect on zooplankton communities. Since sediment samples from RF−PA were taken one week after glyphosate application, the microbes in RF−PA sediment may suffer from this herbicide, resulting in a lower number of zooplankton than in RF−NPA.

## Conclusions

In our work, we have increased knowledge of the diversity of zooplankton hatching from different rice field sediments. We found that, although the number of taxa of zooplankton between RF−NPA and RF−PA was close, species compositions of zooplankton were different, indicated by the Sørensen–Dice index and NMDS result. RF–NPA also had a higher species diversity index and evenness index than RF–PA. In addition, the incubation time potentially influenced species richness and abundance of zooplankton. The highest species richness and abundance of zooplankton in RF–NPA occurred on day 24. However, some zooplankton species were found only on day 6, such as *M.micrura* for RF–PA and nauplius of copepods for RF–NPA. In RF−PA, *L.bulla* and *L.hamata* had a low number of individuals; moreover, *Le.diaphanus* appeared with delayed hatching. Our results indicate that RF−NPA has more diversity than the RF−PA, according to the diversity indices which might be the effect of herbicide application in the sampling year. We also suggest that the hatching of resting eggs is a potential method for the study of zooplankton diversity in temporary habitats.

## Supplementary Material

C95070D6-C1FA-5749-A0C2-702EB1459D46 10.3897/BDJ.11.e106418.suppl1 8251720 Supplementary material 1 Diversity indices and species richness of zooplankton Data type index Brief description Diversity indices and species richness of zooplankton amongst ten sampling stations of RF–NPA and RF–PA. File: oo_885909.xlsx https://binary.pensoft.net/file/885909 Nattaporn Plangklang and Sujeephon Athibai

CEA04620-A2AD-581F-8291-6938810A898A 10.3897/BDJ.11.e106418.suppl2 Supplementary material 2 Similarity index of zooplankton Data type index Brief description Similarity index of zooplankton between sampling stations within rice fields. File: oo_885910.xlsx https://binary.pensoft.net/file/885910 Nattaporn Plangklang and Sujeephon Athibai

## Figures and Tables

**Table 1.** Species list of zooplankton and their abundance in RF−NPA and RF−PA amongst five incubation times (−: absent).

**Scientific Name**	**RF−NPA**					**RF−PA**				
	Day 6	Day 12	Day 18	Day 24	Day 30	Day 6	Day 12	Day 18	Day 24	Day 30
Rotifers										
1. *Cephalodellaforficula* (Ehrenberg, 1830)	3	−	1	−	−	−	−	−	−	−
2. *C.gibba* (Ehrenberg, 1830)	−	−	−	2	1	−	2	5	−	−
3. *Colurellaobtusa* (Gosse, 1886)	−	1	3	11	1	−	26	31	9	1
4. *Euchlanisincisa* Carlin, 1939	−	−	−	−	−	−	−	1	15	4
5. *Lecanearcula* Harring, 1914	−	2	3	1	−	−	−	−	−	−
6. *L.bulla* (Gosse, 1851)	4	81	355	325	172	5	16	4	−	1
7. *L.closterocerca* (Schmarda, 1859)	3	−	−	72	10	−	163	408	171	16
8. *L.haliclysta* Harring & Myers, 1926	−	−	−	−	−	−	−	−	2	2
9. *L.hamata* (Stokes, 1896)	7	98	198	164	50	5	74	96	70	4
10. *L.inopinata* Harring & Myers, 1926	−	−	−	−	−	2	−	−	−	−
11. *L.signifera* (Jennings, 1896)	−	−	−	−	−	1	−	−	−	−
12. *L.tenuiseta* Harring, 1914	−	3	18	23	6	−	20	38	46	9
13. *L.undulata* Hauer, 1938	−	−	−	−	−	−	−	−	3	1
14. *Lepadellapatella* (Müller, 1773)	−	9	5	5	−	1	−	−	−	−
15. *Sinantherinaspinosa* (Thorpe, 1893)	−	−	−	1	1	−	−	3	−	−
16. *Testudinellapatina* (Hermann, 1783)	−	−	2	1	1	−	−	−	−	−
Cladocerans										
17. *Ephemeroporusbarroisi* (Richard, 1894)	−	−	9	43	48	−	−	−	−	−
18. *Ilyocryptusspinifer* Herrick, 1882	−	−	−	−	7	−	−	−	−	−
19. *Karualonakarua* (King, 1853)	−	−	−	−	−	−	2	6	8	12
20. *Leberisdiaphanus* (King, 1853)	2	5	5	23	35	−	−	−	1	1
21. *Leydigiaciliata* (Gauthier, 1939)	−	−	1	1	−	−	−	−	−	−
22. *Macrothrixtriserialis* Brady, 1886	2	5	17	39	11	−	−	−	−	−
23. *Moinamicrura* Kurz, 1874	−	−	−	−	−	1	−	−	−	−
Copepods										
24. Nauplius larva	1	−	−	−	−	−	−	−	−	−
Total species richness	7	8	12	14	12	6	7	9	9	10
Total abundance (individuals)	22	204	617	711	343	15	303	592	325	51

**Table 2.** The Shannon–Wiener diversity index (H´) and Pielou's evenness index (*J*) of zooplankton community from RF–NPA and RF–PA sediments.

**Diversity indices**	**Rice field**	**Incubation time**					**Total data**
		**Day6**	**Day12**	**Day18**	**Day24**	**Day30**	
H´	RF–NPA	1.831	1.172	1.116	1.632	1.566	1.521
	RF–PA	1.542	1.289	1.040	1.370	1.845	1.339
*J*	RF–NPA	0.941	0.563	0.449	0.618	0.630	0.536
	RF–PA	0.861	0.662	0.473	0.623	0.801	0.483

**Table 3.** Summary of estimates of total species richness (± SE) for RF–NPA and RF–PA.

**RF**	**Species observed**	**Chao1**	**Jackknife1**	**Jackknife2**	**Bootstrap**
RF–NPA	17	17.09 ± 0.38	17.98 ± 0.98	14.24	18.11 ± 0.95
RF–PA	16	18.61 ± 3.48	19.92 ± 2.40	20.94	17.96 ± 1.34
